# Managing Pancreatic Adenocarcinoma: A Special Focus in MicroRNA Gene Therapy

**DOI:** 10.3390/ijms17050718

**Published:** 2016-05-13

**Authors:** Marta Passadouro, Henrique Faneca

**Affiliations:** 1 Center for Neuroscience and Cell Biology, University of Coimbra, 3004-504 Coimbra, Portugal; passadouro.marta@gmail.com; 2 Department of Life Sciences, Faculty of Science and Technology, University of Coimbra, 3004-517 Coimbra, Portugal

**Keywords:** pancreatic cancer, pancreatic ductal adenocarcinoma, microRNAs, gene therapy, microRNA-based therapeutics

## Abstract

Pancreatic cancer is an aggressive disease and the fourth most lethal cancer in developed countries. Despite all progress in medicine and in understanding the molecular mechanisms of carcinogenesis, pancreatic cancer still has a poor prognosis, the median survival after diagnosis being around 3 to 6 months and the survival rate of 5 years being less than 4%. For pancreatic ductal adenocarcinoma (PDAC), which represents more than 90% of new pancreatic cancer cases, the prognosis is worse than for the other cancers with a patient mortality of approximately 99%. Therefore, there is a pressing need for developing new and efficient therapeutic strategies for pancreatic cancer. In this regard, microRNAs not only have been seen as potential diagnostic and prognostic molecular markers but also as promising therapeutic agents. In this context, this review provides an examination of the most frequently deregulated microRNAs (miRNAs) in PDAC and their putative molecular targets involved in the signaling pathways of pancreatic carcinogenesis. Additionally, it is presented a summary of gene therapy clinical trials involving miRNAs and it is illustrated the therapeutic potential associated to these small non-coding RNAs, for PDAC treatment. The facts presented here constitute a strong evidence of the remarkable opportunity associated to the application of microRNA-based therapeutic strategies as a novel approach for cancer therapy.

## 1. Introduction

Pancreatic cancer (PC) is known to be one of the most deadly cancers, with a median survival inferior to 6 months and approximately 2% of survival within 5-years after diagnosis [1]. Despite its moderate incidence when compared to other carcinomas, PC accounts for the highest mortality rate by far, the survival associated to it showing the slightest improvement over the past 30 years [2]. It holds the fourth position in the USA and EU in deaths related to cancer, being only surpassed by colorectal, breast and lung and bronchus cancers [2,3]. Pancreatic cancer still remains as an unsolved therapeutic challenge for science despite all efforts carried out to improve current treatments. Only minor significant advances have been achieved to unravel key mechanisms of PC, and always with a modest clinical impact [4]. Besides, most cases are still diagnosed at advanced stages of the disease mainly due to the lack of early symptoms or to symptoms resembling other diseases, consequently no improvement in survival prognosis being achieved with current diagnostic approaches. Most patients present locally advanced or metastatic disease and thus are not eligible for curative surgery [5].

Pancreatic ductal adenocarcinoma (PDAC) is the most predominant type of PC, accounting for more than 90% of pancreatic cancers [6]. PDAC is a very aggressive malignancy that is associated to a very low 5-years survival rate of approximately 2%. Early and belligerent metastization to distant organs also characterizes this type of tumor, being one of its most hostile features. PDAC is clinically classified into three stages regarding the treatment strategy, namely, resectable, unresectable locally advanced and metastatic, each of them with different therapeutic approaches. Surgical resection of the primary tumor still holds the major hope for patients, although candidates to this surgery represent a very low percentage of all patients, and often allows to remove only a small part of the tumor [7]. Current chemotherapy is frequently insufficient and controversial in the treatment of inoperable PDAC patients [8]. Although many studies point towards the use of a cocktail of different chemotherapeutic agents in combinations with radiation treatments, gemcitabine is the frontline therapy with the better outcome in unresectable tumor cases, representing for now the most prominent strategy in terms of overall survival [8]. Life span for PDAC patients is largely unsatisfactory, therefore the development of new and more efficient therapeutic strategies involving different therapeutic molecules, such as microRNAs (miRNAs), is urgently required. miRNAs are small RNA molecules that post-transcriptionally regulate gene expression, consequently having a fundamental regulatory activity in processes such as cell cycle, cellular differentiation, survival, proliferation and apoptosis. Each type of cancer, including PDAC, presents a miRNA signature, characterized by abnormal miRNA expression levels. Therefore, restoring appropriate miRNA levels in tumoral cells may be vital to improve PDAC treatment.

## 2. Current Therapeutic Strategies for Pancreatic Ductal Adenocarcinoma (PDAC)

### 2.1. Resectable Surgery

Despite the important advances that have been made towards the development of better cancer treatments, resectable surgery still stands as the most efficient strategy for cancer patients, being the surgeries mainly performed through the techniques of robot assisted, laparoscopic, or the traditional open approach. Removal of primary tumor and adjacent tissue permits to cure more patients than any other form of cancer therapy, as it permits to remove almost 100% of tumor cells, while other therapeutic strategies only affect a smaller percentage of tumor cells. Nevertheless, even with a resection surgery, only 15%–20% of these PDAC patients survives for 5 years after diagnosis [9]. Therefore, due to elevated rates of failure following curative resection, effective adjuvant strategies became an urgent need in order to improve long-term survival of the patients.

### 2.2. Radiotherapy

Surgery and radiation are considered to be local treatments as they directly target the tumor in a specific area of the body. Nevertheless, more than 80% of the patients with PDAC cannot be submitted to surgery at the time of diagnosis, and half of these patients have already developed distant metastasis, making this type of cancer one of the most difficult to handle from a clinical point of view [10].

Radiotherapy appears as viable option for the treatment of cancer, as it is a non-invasive treatment and patients usually present a faster and easiest recovery. Near 52% of cancer patients undergo radiotherapy at least once during their treatment course [11]. The mechanism underlying radiotherapy is irradiation with high-energy radiation (X-rays, γ rays and fast-moving charged particles like electrons and protons) damaging intracellular components like DNA, thus leading to cell death [12]. However, radiation lacks specificity, as it damages not only the solid tumors but also healthy tissue, suggesting that other complementary tools may be of extreme importance to overcome this obstacle. For this purpose, many studies are being addressed. For instance, Babaein and Ganjalikhani suggest the application of nanoparticles as radio sensitizer in order to make tumor cells more susceptible to radiation. These nanoparticles are designed to increase the efficiency of radiotherapy and reduce the damage of healthy tissues, being considered a new promising strategy to radiotherapy [13].

Considering PDAC, performance status, tumor size and cachexia of the patient have a significant influence on the outcome of (neo) adjuvant radiotherapy, making it a limited option for the treatment of these patients, thus paving the way for other therapeutic strategies, such as chemotherapy. Nevertheless, many times both therapeutic strategies are used in combination, chemo-radiation, in order to ameliorate the patients status and decrease side effects [10].

### 2.3. Chemotherapy

Chemotherapy is still the golden standard treatment for the vast majority of unresectable tumors, either alone or in combination with other treatments. Nowadays, a considerable number of drugs are available for the treatment of the innumerous cancer types, and many others are in study phases, waiting to be approved for clinical application.

Nevertheless, no treatment has had a significant impact on pancreatic cancer so far and most of the currently used drugs are to relief and control the patient symptoms (Table 1). Up to now, one of the standard of care for patients with unresectable pancreatic cancer is fluorouracil (5-FU) combined with radiotherapy, which was introduced in 1969 after a trial study indicating an improved median survival time from 6.3 to 10.4 months, when compared to radiotherapy alone [14]. Gemcitabine is the other standard treatment that was introduced by Burris *et al.* [15] in the late 1990s after a comparative study of the efficacy of gemcitabine against 5-FU. Despite, Burris and colleagues did not registered a significant increase in overall survival time (5.65 months for gemcitabine against 4.41 months for 5-FU), patients treated with gemcitabine exhibited a better clinical response, resulting in a significant improvement on patients symptoms, 23.8% against 4.8% obtained with 5-FU [15]. Along the years, many studies have questioned which of these two drugs achieved a better outcome for PDAC patients. However, none of these studies could fully elucidate this issue, since both drugs exhibit similarly low survival rates, proving that the treatments for pancreatic cancer have been failing to improve long-term survival.

The search for new drugs that could improve patient’s survival and surpass acquired chemoresistance led to the discovery of paclitaxel as a possible chemotherapeutic agent that could be administrated along with gemcitabine [18]. More recently, a new therapeutic strategy, consisting of human albumin nanoparticles bound to paclitaxel, nab-paclitaxel (ABRAXANE), was developed in order to promote an efficient delivery of paclitaxel into tumor cells [19]. Promising data have emerged from a pilot Phase I/II study where advanced PC patients were submitted to a chemotherapeutic regiment of a combination of nab-paclitaxel and gemcitabine. The median survival rate registered was 8.5 months, and the overall response rate was 23% [17].

Another recent chemotherapeutic regiment with proven efficacy in metastatic solid cancers, such as colon and pancreatic cancer, is FOLFIRINOX, which is the combination of several drugs: fluorouracil, irinotecan, oxaliplatin and folinic acid. Single-agent oxaliplatin has low activity in many tumours, nevertheless, when combined with fluorouracil or irinotecan, a synergistic effect is observed in the treatment of solid tumors [20,21]. Due to the relatively non-overlapping toxicities of fluorouracil, folinic acid, irinotecan, and oxaliplatin, a regimen combining these agents was studied in a phase I clinical trial showing significant responses in patients with advanced pancreatic cancer [16,21]. In 2011, Thierry Conroy and colleagues performed this clinical trial in order to elucidate the therapeutic potential of FOLFIRINOX in comparison with a gemcitabine monotherapy, in patients with a low severe status of illness [16]. They observed a significant increase in patient’s survival, with a median overall survival of 11.1 months in the FOLFIRINOX group as compared to 6.8 months in the gemcitabine group. Moreover, the objective response rate was 31.6% in the FOLFIRINOX group *versus* 9.4% in the gemcitabine group. However, patients of the FOLFIRINOX group exhibit more severe side effects, revealing an undesirable toxicity. Nevertheless, this drug regiment was considered to be a valuable option for the treatment of patients with metastatic pancreatic cancer and to have good performance status [16].

Pancreatic cancer is a very heterogeneous and highly complex disease, with a wide variety of activated tumor pathways. Although patients’ survival rates treated with these therapeutic regimens remain largely unsatisfactory, these drugs still hold the best treatment for pancreatic cancer. These data point out the urgent need for additional investigation towards the discovery and development of new and efficient multitargeted therapeutic strategies for PC, such as those involving microRNAs.

## 3. MicroRNAs

The continuous search for new molecular targets for cancer therapies has allowed the identification of several major cellular regulators involved in carcinogenesis, such as miRNAs.

MicroRNAs were first identified in *Caenorhabditis elegans* in the beginning of the 1990s, when Lee *et al.* [22] discovered that a small 22 nucleotide RNA sequence, lin-4, could negatively regulate the level of lin-14 protein. It was suggested for the first time that this sequence was responsible for regulating translation via an antisense RNA–RNA interaction, since it bound in a complementary way to the 3’ untranslated region (UTR) of lin-14 mRNA. miRNAs are small endogenous non-coding RNAs with 20 to 22 nucleotide length, but with a powerful task of modulating gene expression in a post-transcriptional manner. miRNAs bind to the target at the 3′ UTRs through imperfect complementarity at multiple sites, inducing translational repression or transcript degradation, depending on the degree of complementarity between miRNA molecule and the target mRNA [23]. Bioinformatic algorithms have estimate that each miRNA can target hundreds of different mRNAs, hypothesizing that a large proportion of the transcriptome is under the control of miRNA regulation [24]. Therefore, miRNAs display extensive regulatory activity in virtually all biological processes such as cell cycle, cellular differentiation, survival, proliferation and apoptosis [25]. Although these tiny molecules are able to regulate a large portion of the genome, Fromm B *et al.* [26] have recently stated that most provably only approximately 30% of the 1,881 human miRBase entries, and approximately 16% of the 7095 metazoan miRBase entries, are strongly supported as *miRNA* genes.

### 3.1. MicroRNAs and Cancer

MicroRNAs can target cancer related genes, therefore they can act either as oncogenes (oncomiR), promoting proliferation and/or repressing apoptosis, or as tumor suppressors, by repressing genes responsible for the maintenance of tumorigenesis [27]. The first reported case of abnormal miRNA expressions was found in B-cell chronic lymphocytic leukemia (CLL) [28]. After that, several genome-wide profiling studies were performed in various solid tumors, such as breast cancer, glioblastoma, hepatocellular carcinoma, lung cancer, colon cancer and endocrine pancreatic tumors, having been identified anomalous microRNA expression patterns in all cancers [29]. Normal and tumor tissues exhibit different miRNA expression profiles, and a specific miRNA signature may be observed for a certain tumor type, which can be used to characterize and identify them [30]. Interestingly, miRNAs were also identified in many human fluids, including human blood stream, as circulating miRNAs, making them a valuable tool as biomarkers for cancer diseases, since patients and control groups could be easily distinguished by analyzing a specific set of miRNAs [31]. Furthermore, specific miRNA expression signatures have been identified not only as characteristic of some cancer subtypes, and therefore useful for tumor classification, but also as feature of cancer prognosis, staging, and response to therapy [29,31,32].

Special attention has been devoted to miRNAs involved in different cellular pathways of high importance to maintain cancer malignancy. Some of these miRNAs are pivotal players in several carcinogenic pathways, revealing a widespread monopoly over tumoral maintenance [29]. For instance, microRNA-21 (miR-21) has attracted the attention of researchers in various fields, and is probably one of the most extensively studied miRNAs [33]. Moreover, miR-21 was identified as the best hit in a number of profiling experiments designed for the detection of miRNAs deregulated in cancer [33]. It was shown to be strongly up-regulated in various types of tumors, including glioblastoma, breast cancer, colon cancer, lung cancer, pancreas cancer, prostate cancer, gastric cancer and hepatocellular carcinoma, acting in some pivotal signaling pathways involved in tumor growth, invasion and chemoresistance, making miR-21 one of the most promising prognostic markers for cancer diagnose [33].

Another example of well-studied miRNAs is the let-7 family, which is frequently downregulated in the vast majority of cancers. Let-7 tumor suppressor activity was found to exert an effect upon two of the most important oncogenic genes, *RAS* (encodes proteins that are involved in kinase signaling pathways) and *c-MYC* (v-myc avian myelocytomatosis viral oncogene homolog) [34,35]. This miRNA’s functions extend from suppression of cell proliferation, to induction of apoptotic signaling pathways and sensitization to chemotherapeutic agents [36,37].

Many different miRNAs can be found deregulated in cancer, some exerting oncogenic roles in signaling pathways that promote tumor growth, increased angiogenesis, stemness, *etc.*, others prevented from regulating normal physiological events. One of the most aggressive characteristics of cancer is the ability of tumor cells to escape from the primary tumor and invade healthy tissues, thus promoting metastasis formation. This event is also supported by the action of oncogenic microRNAs. Abba M. and colleagues reviewed the impact of miRNAs in the regulation of matrix metalloproteinase, which are enzymes responsible for the breakdown of collagen Type IV, thus responsible for extracellular matrix and tissue remodeling [38]. However, these proteins are also endorsed in cancer progression, epithelial to mesenchymal transition (EMT) and metastization, being the normal physiological processes disrupted. The review performed by Abba M and colleagues, comprising data from 55 different studies, showed a group of 13 miRNAs that target the matrix metalloproteinases (MMPs) MMP-2 and MMP-9 in a large variety of cancers types. Amongst them, miR-10b, miR-21, miR-125b, miR-138 and miR-181b showed that the regulation of these MMPs, and thus the associated invasion of normal tissue by tumor cells and the establishment of novel metastasis, are strongly determined by the action of a multiplicity of miRNAs [38].

### 3.2. Deregulated MicroRNA in PDAC

PDAC shares a common feature with other solid tumors: an abnormal expression of miRNAs, usually implicated in several supportive mechanisms of oncogenesis. Studies based on high-throughput microarray technologies have been performed using available *in vitro* models as well as tumor samples, excised from PDAC patients, in order to establish a common expression pattern for this malignancy, and thus a tumor miRNA signature for pancreatic cancer [39,40,41]. Nevertheless, when it comes to identify a large group of differentially expressed miRNAs in PDAC, consensus among research groups has been an arduous issue, since numerous parameters, such as differences in measurement platforms and laboratory protocols, small sample sizes as well as variability among samples, can strongly influence the attained results and consequently justify the absence of reproducibility. Nevertheless, there is a relatively small group of miRNAs (Table 2) with deregulated expression that is consistent across different studies involving PDAC samples [39,40,41,42,43]. For instance, Ma M. *et al.* [42] reviewed eleven miRNA profile studies in PDAC, reporting 439 miRNAs differentially expressed, that gathered a total of 538 tumors and 206 noncancerous control samples, showing some consistent miRNAs that are deregulated in PDAC samples.

Exhaustive investigation has been devoted to miRNAs to further clarify their role in signaling pathways responsible for supporting tumor cells proliferation, survival and metastasis in pancreatic cancer. The here highlighted miRNAs are profoundly embedded in some of the most important and mentioned hallmarks of PDAC, such as invasion, migration and metastasis formation. Therefore, this group of miRNAs, which will be reviewed below, can be probably associated to the known aggressive profile of PDAC, such as the resistance to the overall available treatments and the highest mortality rate, providing a more real picture of the true nature of this belligerent type of cancer.

#### 3.2.1. Up-Regulated MicroRNAs in PDAC

##### miR-10b

Regarding miR-10b up-regulation in pancreatic cancer, Preis M. and colleagues, analyzed several samples of PDAC and found that miR-10b was one of the most frequently and consistently overexpressed microRNAs [66]. Their data also suggested that lower levels of miR-10b were correlated with a better response to neoadjuvant therapy, higher probability of surgical resection, delayed time to metastasis, and increased survival [66]. More recently, a study revealed some possible mechanistic properties of miR-10b in promoting invasiveness in pancreatic cancer, as Tat-interacting protein 30 (TIP30) was identified as its direct target. MiR-10b was shown to suppress TIP30 expression, which in turn enhances epidermal growth factor receptor (EGFR) signaling, promotes epidermal growth factor (EGF)—transforming growth factor β (TGF-β) cross-talk and increases the expression of EMT-inducing genes, while reducing the expression of several metastasis-suppressing genes [44].

##### miR-100

Distinct roles can be attributed to miR-100 in different cancers, making it a quite contradictory microRNA, as it can behave either as an oncogene or a tumor suppressor gene, depending on the tumor type. Chen J. and colleagues reviewed this contradictory role of miR-100 in a variety of cancers, reporting several studies where this microRNA can display either oncogenic or tumor suppressor features [67]. For instance, in breast cancer miR-100 was found to be downregulated and was related with progressive pathological feature and poor prognosis in patients. Reestablishment of miR-100 expression levels led to tumor growth inhibition by strongly reducing IGF2 (insulin-like growth factor 2) expression, a known oncogene [68]. Concerning pancreatic ductal carcinoma, miR-100 was described to be up-regulated in patients and in human pancreatic cancer stem cells, demonstrating its extensive importance in the tumorigenic process of PDAC [69,70]. Moreover, it was reported that metastatic pancreatic cell lines present a much greater expression of miR-100 than non-metastatic cell lines [45]. A functional link was established between miR-100 and IGFR1 (insulin-like growth factor 1 receptor), known to be involved in the proliferation mechanisms in cancer and to control the ability of pancreatic cancer cells to metastasize *in vivo*. It was observed that the IGFR1 expression levels decrease after transfection of pancreatic cancer cells with miR-100 inhibitors [45]. Nevertheless, the consequences of miR-100 overexpression in PDAC are still poorly understood.

##### miR-155

miR-155 is known to play a crucial role in the post-transcriptional regulation of TP53INP1 (tumor protein 53-induced nuclear protein 1), which is under the direct control of p53, a tumor suppressor gene. TP53INP1 induces cell cycle arrest and apoptosis, and its expression is lost in early stages of pancreatic cancer. Gironella and colleagues observed that miR-155 is responsible for repressing TP53INP1 expression and that the restoration of TP53INP1 levels is in accordance with the regression of tumorigenic features of pancreatic cancer [46]. Furthermore, miR-155 was also shown to be involved in the control of invasiveness and migration ability of pancreatic tumoral cells by modulating the STAT3 (signal transducer and activator of transcription 3) pathway through the reduction of SOCS1 (suppressor of cytokine signaling 1) expression levels [47]. Additionally, abnormal levels of miR-155 were detected in noninvasive precursor lesions, a premature stage of pancreatic cancer, and increased oncogenic activity of miR-155 was related with poorer survival chances in PDAC patients, making this microRNA a fundamental biomarker in differentiation of this malignancy and a predictor for patient outcome [71,72].

##### miR-196a

Biological relevance of miR-196a in pancreatic cancer is still largely unclear, despite being mentioned in several genome-profiling studies as one of the most differentially expressed microRNAs in pancreatic cancer. Recently, Huang and colleagues suggested that the nuclear factor κB-inhibitor α (NFKBIA), an inhibitor of the NF-κB transcription factor, which is implicated in the progression of pancreatic cancer, is a target of the miR-196a in PDAC [48]. They registered increased levels of miR-196a in four different pancreatic cell lines and enhanced expression of NFKBIA after miR-196a down-regulation, which promoted inhibition of migration, suggesting a direct regulation mechanism of miR-196a in migratory ability of pancreatic tumor cells. Moreover, miR-196a was also pointed as an important modulator in processes such as apoptosis, invasion, and proliferation in pancreatic cancer cells [49]. miR-196a role was monitored *in vitro* and *in vivo* for functional contributions to apoptosis, invasion, and growth in these pancreatic cancer cells. It was demonstrated that cells displaying higher content of miR-196a reveled a decreased ING5 (inhibitor of growth protein 5) expression that was associated to a reduced apoptosis, enhanced invasion and growth of this type of tumor. When cells were transfected with a miR-196 inhibitor, the opposite effect was observed [49]. Regardless of all these evidences relatively to the oncogenic role of miR-196a in pancreatic cancer, as far as we know, no attempt has been made to use this promising microRNA as a therapeutic strategy for the treatment of PDAC.

##### miR-21

miR-21, as in many other type of malignancies, is strongly up-regulated in PDAC [73,74,75]. miR-21 has proved to be one of the miRNAs with most significant impact in PDCA, being related to metastatic degree or proliferation capacity [73,76]. This microRNA has demonstrated to be enrolled in cell proliferation, survival and gemcitabine resistance in pancreatic ductal adenocarcinomas [50,77], and most importantly, it is detected in early pancreatic lesions known to be precursor of pancreatic cancer [78]. We and others have shown that miR-21 regulates the function of several tumor suppressor genes such as *PTEN*, a phosphatase and tensin homolog gene that negatively regulates the PI3K/Akt pathway [74,75,77,79]. PTEN is a tumor suppressor factor playing a dual protein/lipid phosphatase activity in the PI3K signaling pathway, which in turn controls major biological processes such as cellular growth, proliferation and protein synthesis, by directly acting as a central negative regulator. Moreover, recent reports suggest complementary roles for miR-21 in pancreatic cancer. For instance, hypoxic microenvironment of pancreatic tumor was shown to regulate miR-21 expression levels through increase of the hypoxia-inducible factor 1-α (HIF-1α) expression, and hypoxic conditions are described as metastasis enhancer [80]. On the other hand, pancreatic tumor cells were reported to improve their ability to invade and metastasize by inducing tumor-associated fibroblasts (TAFs) to express miR-21 [81]. Taken together, these data show that miR-21 is a promising dual target, both in tumor cells and TAFs.

##### miR-221/222

miR-221 was found to be up-regulated in pancreatic cysts with malignant potential and to drive invasive cancer, demonstrating that miR-221 aberrant expression is also an early event in the development of pancreatic cancer [82]. This miRNA is part of a gene cluster also expressing miR-222 and both have a predicted target sequence in the 3’ untranslated region (UTR) of several targets acting as their strong repressors [52,53,54,55]. miR-221/222 were reported in several studies to be a key player in diverse pathological pathways in PDAC such as cell proliferation, survival, migration, invasion, metastasis, and acquisition of the EMT phenotype [52,53,54,55]. These events have been demonstrated to be partially a consequence of the down-regulation of the miR-221/222 target, cyclin-dependent kinase inhibitor 1B (p27^Kip1^) [53,54]. Moreover, miR-221 was described to be a crucial element in these pathological events in PDAC by regulating the platelet-derived growth factor (PDGF) signaling cascade [52]. Some other mRNA targets of miR-221 were highlighted by Sarkar S *et al*. [54] revealing that the inhibition of miR-221 could decrease the proliferative capacity of the pancreatic cancer cells by rescuing the tumor suppressor activity of PTEN, p27^kip1^, p57^kip2^ (cyclin-dependent kinase inhibitor 1C), and PUMA (p53 upregulated modulator of apoptosis), which are well-known tumor suppressors. Recently, the metallopeptidase inhibitor 2 (TIMP-2), a tissue inhibitor of metalloproteinases, was also identified as a direct target of miR-221/222 [55]. The overexpression of these microRNAs were associated to an oncogenic activity in pancreatic cancer by promoting the expression of MMP-2 and MMP-9, consequently inducing cancer cell invasion, through the down-regulation of TIMP-2 [55].

##### miR-23a

Recent studies with miR-23a clearly relate its activity with metastatic potential of tumoral cells. miR-23a is located in the miR-23a/24/27a cluster, which functions as an oncogenic miRNA cluster in several human cancers, and was detected to be highly overexpressed in gastric cancer, being associated with down-regulation of metallothionein 2A (MT2A), consequently promoting growth of malignant cells [83]. An inverse correlation between miR-23a and the tumor suppressor interferon regulator factor 1 (IRF1) expression as well as the *E*-cadherin expression was also identified, demonstrating new targets for this miRNA in gastric cancer [84,85]. In the case of the colorectal cancers, miR-23a expression was found to be more restricted to its invasive form and the suppression of miR-23a was correlated with inhibition of cell motility, cell migration, and invasion. Furthermore, miR-23a was detected among the most highly expressed miRNAs in colorectal cancer stem cells (CCSCs) [86,87]. In both cases, colorectal cancer cells and CCSCs, the overexpression of miR-23a was associated with inhibition of the metastasis suppressor 1 (MTSS1), consequently promoting cell migration and invasion [86,87]. The knockdown of miR-23a in colorectal tumor cells was also able to allow the occurrence of 5-FU-induced apoptosis *in vitro* through the apoptotic protease activating factor 1 (APAF-1)/caspase-9 apoptotic pathway, consequently overcoming the initial resistance to this chemotherapeutic regiment [88]. Regarding the pancreatic cancer, Liu N and colleagues reported that miR-23a was differentially up-regulated in six *in vitro* models of pancreatic cancer, highlighting the oncogenic role for this microRNA in PDAC [56]. These authors correlated the overexpression of miR-23a with the reduced APAF-1 expression levels that promoted cell proliferation and repressed apoptosis [56]. Surprisingly, Pellegrino and colleagues reported that, in breast cancer, low levels of miR-23b promotes tumor growth and induces cell motility and invasion, thus, down-regulation of this miRNA seems to assume a tumorigenic stimulus in breast cancer [89]. Herein, a fragile equilibrium in the amount of miRNA in the cell appears to be the key for normal function in healthy cells. In summary, this miRNA displays a pivotal role in metastasis and chemoresistance.

#### 3.2.2. Down-Regulated MicroRNAs in PDAC

##### miR-148a

Similar to other deregulated miRNAs in PDAC, miR-148a was also described as aberrantly under expressed in hepatocellular carcinoma, gastric cancer and non-small lung cancer, being associated with more aggressive features and poor survival rates [90,91,92]. The major pathway in which miR-148a seems to play a pivotal role in malignancy control are intrinsically related to the EMT, predominantly acting as a metastasis suppressor [90,91,92]. The inhibition of EMT through miR-148a action is partially attributed to the downregulation of vimentin and to the up-regulation of *E*-cadherin. In addition, miR-148a was also found to inhibit cancer metastasis by suppressing TGFβ-induced EMT through SMAD2, its direct functional target in human gastric cancer [93]. Moreover, this miRNA was shown to attenuate cancer stem cells (CSCs)-like properties through the inhibition of TGF-β/SMAD2 signaling pathway upon treatment with Glabridin [94]. Importantly, miR-148a gene expression inactivation was shown to be a consequence of DNA hypermethylation in PDAC-derived cell lines and PDAC samples, compared with adjacent samples of non-pathologic tissue [95]. Moreover, evidences of aberrant hypermethylation of the miR-148a coding region were reported to occur early in human PDAC precursor pancreatic intraepithelial neoplasia (PanIN) lesions [95]. Therefore, this epigenetic event is deeply involved in the premature loss of miR-148a expression in pancreatic cancer [95]. In 2011, Sven-T Liffers and colleagues observed, through luciferase based reporter assays, that the protein phosphatase CDC25B (cell division cycle 25 B) was a candidate target of miR-148a [57]. Moreover, using an *in vitro* model based on lentiviral-mediated stable miR-148a overexpression in the IMIM-PC2 pancreatic carcinoma cell line, they demonstrated that miR-148a overexpression had an inhibitory influence on the growth potential of pancreatic cancer cells [57]. More recently, two oncogenic genes, *cholecystokinin-B receptor* (*CCKBR*) and *B cell lymphoma 2* (*Bcl-2*), were found to be under the post-transcriptional regulation of miR-148a. This miRNA was found not only to inhibit the proliferation of pancreatic cancer cells, but also to promote cells apoptosis *in vitro* through the suppression of CCKBR and Bcl-2 expression [58]. On the other hand, another research group observed that there was no dramatic effect of miR-148a on cell proliferation and cell chemo-sensitivity in four PDAC cell lines [96]. They evaluated cell sensitivity to gemcitabine and concluded that no correlation exists between miR-148a expression levels and intrinsic sensitivity to gemcitabine, in their experimental conditions. Although these authors observed that this miRNA was down-regulated in PDAC, they suggested that miR-148a had a weak therapeutic potential [96]. Nevertheless, in recent years several research groups have demonstrated that miR-148a has a crucial role in PDAC carcinogenesis and that its overexpression results in a significant antitumor activity in different pancreatic cancer models [57,58,59,96].

##### miR-217

Emerging evidences points towards a tumor suppressor function of miR-217 in several types of cancer. Low cellular levels of this miRNA were associated with improved invasion ability, increased cell motility and cell proliferation in both renal cell carcinoma and hepatocellular carcinoma [97,98]. Moreover, it was described that miR-217 downregulation was associated with drug-resistance in chronic myelogenous leukemia [99]. The molecular mechanisms involving miR-217 were investigated in PDAC by Wu-Gan Zhao and colleagues, who analyzed this miRNA expression profile in normal and tumoral tissue from patients as well as in PDAC cell lines [60]. These authors reported a different profile in healthy and malignant samples, being miR-217 significantly down-regulated in PDAC tissues and cell lines, when compared to that obtained in normal pancreatic tissue [60]. Furthermore, a significant suppression of cell growth was observed after ectopic expression of miR-217, both *in vitro* and *in vivo*, which was inversely correlated with KRAS (kirsten rat sarcoma viral oncogene homolog) expression, due to direct post-transcription regulation [60]. Moreover, the expression of miR-217 had also the ability to affect other downstream molecular effectors, indicating a regulatory role in KRAS signaling pathway [60]. Reduction of KRAS protein expression in a miR-217 dependent manner promoted the decline of anchorage-independent colony formation in PDAC cells. Remarkably, after *in vivo* xenograft treatment with miR-217 expression vector it was observed a decrease of tumor growth, revealing a therapeutic potential for PDAC [60]. Adding to these findings, Deng S. and colleagues demonstrated that miR-217 is downregulated in pancreatic cancer and was negatively correlated with sirtuin 1 (SIRT1), its direct target. Moreover, the restoration of higher levels of miR-217 prompted mesenchymal to epithelial transition (MET) in TGF-β1 (transforming growth factor beta 1) treated PC cells, unveiling a strong link of this miRNA with late tumor stage, lymphatic invasion, vascular infiltration and distant metastasis [61].

##### miR-34a

miRNA-34a is probably one of the best examples of thoroughly studied miRNAs that are down-regulated in several types of cancer, including PDAC. Hu Q. and colleagues demonstrated that some of the molecular targets that are regulated by miR-34a in pancreatic cancer are E2F3 (E2F transcription factor 3), Bcl-2, c-MYC and cyclin D1 [62]. These authors showed, in a xenograft model of PDAC, that the intracellular delivery of this miRNA induces cell cycle arrest and apoptosis, and suppresses the tumor cell migration, as it will be discussed further ahead as an example of a targeted therapy for PDAC [62]. Similarly, ectopic expression of miR-34a seems to exert an inhibitory effect in the Notch-1 signaling pathway, another known molecular target for this miRNA, restoring apoptosis in pancreatic cancer cells [63].

miRNA-34a has also been reported to account for the regulation of pancreatic cancer stem cells features, such as self-renewal capacity, consequently representing an important tumor suppressor to fight tumor-initiating cells in PDAC [100]. These findings strongly suggest that miR-34a can exert a pivotal role as a tumor-suppressor in pancreatic cancer therapies.

##### miR-375

In the past few years, miR-375 mechanisms in PDAC have been studied, since it is considered one of the most consistently downregulated miRNAs in pancreatic cancer. However, a limited number of studies on pancreatic cancer have focused on the targeting and on the clinical and prognostic significance of miR-375. A large study, involving the analysis of miR-375 expression in normal pancreatic tissue and tumor samples of PDAC, identified miR-375 as candidate with a strong potential for future clinical applications [101]. It was demonstrated that miR-375 might be used to classify normal, chronic pancreatitis and cancerous tissues, allowing discriminating between neoplastic and non-neoplastic processes in pancreatic cancer. In other types of tissues, miR-375 is also described as an important mediator of normal cellular function. For instance, in breast cancer it was identified as targeting short stature homeobox 2 (SHOX2), readily mediating EMT suppression [102]. In addition, epigenetic silencing of miR-375 in HER2-positive breast cancer conferred resistance to trastuzumab treatment, which is the targeted therapy for this disease [103]. Ye X. and colleagues demonstrated that the overexpression of miR-375 restored the sensitivity of HER2-positive breast cancer cells to trastuzumab, most probably due to down-regulation of IGFR1 [103]. Corroborating the same antitumorigenic profile, a study performed in non-small-cell lung cancer (NSCLC) also found miR-375 significantly down-regulated [104]. Moreover, the authors showed that this miRNA could be an important biomarker for survival, as patients with low miR-375 expression had worse overall survival rates than those with high miR-375 expression. Overall, miR-375 is involved in the suppression of core hallmarks of cancer, such as cell growth, invasion, migration, metastasis and proliferation, by targeting several important oncogenes like *AEG-1* (*astrocyte elevated gene-1*), *YAP1* (*yes-associated protein 1*), *IGFR1* and *PDPK1* (*3-phosphoinositide-dependent protein kinase 1*), thus making it an encouraging target in many antitumor strategies [105].

Indeed, miR-375 was first identified as a pancreatic islet-specific miRNA that regulates the glucose-induced insulin secretion, consequently being an important participant in glucose homeostasis by controlling the growth and morphogenesis of the pancreatic islet [105]. Later this miRNA was recognized as a down-regulated miRNA in pancreatic malignant cells [105]. Several studies search for the impact of this down-regulation in PDAC, for instance, Jian Zhou and colleagues provided data correlating miR-375 restoration levels with induced apoptosis and abrogation of cell proliferation *in vitro* [106]. The low levels of this miRNA was also associated with lymph nodes metastasis formation and advanced stage of the disease [105]. One of the potential mechanisms involved in miR-375 action is the repression of the PDPK1 expression, consequently promoting a decrease in the tumorigenicity of pancreatic cells through the regulation of the Akt signaling pathway [65]. Inhibition of PDPK1 by miR-375 includes the inhibition of cell proliferation and the induction of apoptosis and cell cycle arrest at G0/G1 phase in PDAC cells [65]. Moreover, the chemo-preventive agent benzyl isothiocyanate (BITC), known for inhibiting the growth of pancreatic cancer cells *in vitro*, was reported to be capable of modulating the levels of miR-375, along with miR-221, in order to diminish the cell viability and sensitize pancreatic tumor cells to its antiproliferative action [107].

## 4. MicroRNA Therapeutic Potential

The discovery of new molecular targets in cancer, such as the deregulated miRNAs, brings with it the need for innovative tools to reach these oncogenic triggers and consequently the beginning of a new era of cancer treatment strategies with an enormous therapeutic potential.

Anti-miRNA oligonucleotides (AMOs) are molecular tools able to induce miRNA silencing either *in vitro* or *in vivo*, since these compounds have the ability to tightly bind and inactivate the miRNA action [108]. Similarly, miRNA replacement therapy, in which the lower endogenous levels of miRNAs are augmented with recourse to oligonucleotide mimics, is another strategy to modulate intracellular miRNA levels [109]. These oligonucleotides constitute an important tool for the manipulation of miRNA function in biological systems, mainly due to their unique characteristics (low size, low immunogenicity, high target affinity). However, several critical hurdles, such as reduced *in vivo* stability, inappropriate biodistribution, lack of cell specificity, disruption and saturation of endogenous RNA machinery and potential side effects, limit proper delivery of miRNA-targeting agents or miRNA mimics into target cells. In order to overcome these barriers and translate miRNA innovations into clinical applicability, appropriate approaches, including oligonucleotides modification and delivery systems, have been designed.

Chemical modifications of oligonucleotides confer resistance to nucleases and increase their binding affinity to their targets, consequently improving their performance [110]. Locked Nucleic Acid (LNA™) nucleosides are a successful example of this type of miRNA expression modulators, consisting of a class of nucleic acid analogues containing one or more LNA nucleotide monomers with a bicyclic furanose unit locked in a RNA-mimicking sugar conformation. This conformational restriction is translated into exceptional hybridization affinity towards complementary single-stranded RNA molecule and its efficiency has been proven both *in vitro* and *in vivo* [111,112,113].

On the other hand, the development of new and efficient delivery systems could contribute to a successful intracellular delivery of AMOs or miRNA mimics into target cells, consequently allowing the successful application of these new therapeutic strategies. With this purpose, several new gene delivery platforms have been developed (Table 3). For instance, in lung cancer, the downregulated miR-29 was subject of microRNA replacement therapy using a cationic liposome-based system, consisting of DOTAP, cholesterol and d-α-Tocopherylpolyethyleneglycol 1000 succinate (vitamin E TPGS), to efficiently deliver miR-29b both *in vitro* and *in vivo* [114]. This strategy not only promoted a reduction in the expression of key miR-29b targets but also in cell growth and clonogenicity of *in vitro* non-small carcinoma cells. In addition, systemic delivery of these lipoplexes containing miR-29b resulted in an increase in the levels of this miRNA in the tumor, consequently downregulating the tumor mRNA targets, and significantly inhibiting tumor growth *in vivo* [114].

Another work demonstrated that exosomes, small endosome-derived vesicles that are secreted by a variety of cell types and tissues, could efficiently deliver let-7a miRNA to EGFR-expressing breast cancer cells *in vivo*. The authors engineered the donor cells to express the transmembrane domain of platelet-derived growth factor receptor fused to the GE11 peptide in order to target EGFR positive breast cancer cells [115]. Exosomes loaded with let-7a miRNA were intravenously administrated to EGFR-expressing breast cancer mouse model, resulting in significant inhibition of tumor growth [115]. The biocompatibility and toxicity profiles of exosomes, which notably are natural carriers of miRNA *in vivo*, support their application as drug delivery systems.

Viral vectors have also been shown to be highly effective in mediating gene therapy against cancer. Oncolytic adenoviruses have been considered highly eligible vehicles for delivery of therapeutic genes to treat cancer due to their tumor-restricted replication capabilities [116]. Wenjia Lou and colleagues, proposed to use oncolytic adenovirus co-expressing miRNA-34a and IL-24 in a hepatocellular carcinoma xenografts mouse model in order to achieve a synergistic antitumoral effect [116]. Their data demonstrated that miRNA-34a and IL-24 could be efficiently co-expressed after transduction with oncolytic adenovirus (AdCN205-IL-24-miR-34a). Moreover, simultaneous expression of miRNA-34a and IL-24 showed no effect on adenovirus replication ability in HCC tumor cells. The obtained results showed that AdCN205-IL-24-miR-34a could dramatically inhibit the tumor cell growth *in vitro*, and resulted in complete tumor regression without tumor recurrence *in vivo* [116].

Many different options have been explored for efficient delivery of miRNA mimics or AMOs into target cells, in order to develop new therapeutic strategies that present higher efficacy and lesser side effects than conventional ones.

### MicroRNA Therapeutic Potential in PDAC

Much work has been devoted to the development of novel miRNA-based therapeutic strategies for application in PDAC (Table 3). For instance, in 2011, an *in vivo* study was performed using a lipid-based nanosystem, for intravenous administration, containing a DNA plasmid encoding miR-34a in conjunction with miR-143/145, to manage PDAC tumorigenicity [117]. The obtained results pointed for a successful microRNA modulation, since the restoration of miR-34a and miR-143/145 cluster levels in cancer cells promoted both pro-apoptotic and antiproliferative effects in pancreatic cancer xenografts. The systemic delivery of this miRNA-coding plasmid mediated by nanovectors resulted in the growth inhibition of both subcutaneous and orthotopic pancreatic cancer xenografts, demonstrating to be a highly effective tool to addressed PDAC [117]. More recently, another miR-34a delivery strategy comprising nanocomplexes containing a tumor-targeting and -penetrating bifunctional CC9 peptide was developed [62]. The authors showed that treatment with these nanocomplexes resulted in increased levels of miR-34a that promoted down-regulation of its target genes, namely *E2F3*, *Bcl-2*, *c-MYC* and *cyclin D1*, and ultimately the cell cycle arrest, apoptosis and migration suppression. Treatment with these nanocomplexes *in vivo* significantly repressed tumor growth and prompted cancer cell apoptosis [62].

On the other hand, we have recently demonstrated that an almost complete abrogation of up-regulated miRNAs in PDAC was obtained by the intracellular delivery of AMOs using a human serum albumin and lipid-based nanosystem [53]. miR-21, miR-221, miR-222 and miR-10b expression levels in PDAC cell lines were thoroughly inhibited, resulting in a significant increase in their target levels. We also showed that the modulation of miR-21 levels in combination with low amounts of the chemotherapeutic drug sunitinib resulted in a strong and synergistic antitumor effect, demonstrating the therapeutic potential of this combined strategy for PDAC [53].

## 5. MicroRNA-Based Gene Therapy Clinical Trials

More than prognostic biomarkers or diagnostic tools, miRNAs are stepping into a new era of therapeutic strategies against numerous diseases. Several biopharmaceutical companies and research groups are working in this new field of microRNA-based therapeutics, in order to develop new products that present potential to reach clinical trials (Table 3). The first case of a miRNA-based gene therapy product achieving a clinical trial was Miravirsen, a product developed by Santaris Pharma, which had already completed a Phase II clinical trial (Table 3). Miravirsen is a LNA-based miRNA inhibitor targeting miR-122, a liver specific miRNA shown to be crucial for the functional infection of Hepatitis C virus, that demonstrated promising results in the performed clinical trials in terms of both therapeutic efficacy and safety [118,119].

Although several key miRNAs were subject of interest in the regulation of cancer malignancy, clinical trials involving miRNA-based gene therapy approaches for this disease were quite scarce. In this regard, new gene therapy strategies have been designed in order to restore normal expression levels of such miRNAs, by incorporating AMOs or miRNAs mimic into target cells. Some miRNAs have demonstrated to be potential clinical targets for cancer therapy, such as let-7, miR-29, miR-21 and miR-34a [121]. Regarding miR-34a, although several cancer processes are controlled by this miRNA, it is specially enrolled in the p53 transcriptional network and its suppression in cancer cells is tightly related to resistance to apoptosis induced by p53-activating agents [122,123,124]. Moreover, this miRNA can act synergistically with conventional cytotoxic therapies in different cancer types, making it a very interesting option for miRNA-based therapies [120].

Mirna Therapeutics, an American company in miRNA therapeutics, developed the first miRNA replacement therapy for application in cancer clinical trials, using miR-34a mimics (MRX34) incorporated into a lipid-based nanoparticle formulation called SMARTICLES (liposomes composed of lipids having anionic and cationic groups) [120]. This miRNA-based product is in a Phase I clinical trial to evaluate the safety, pharmacokinetics and pharmacodynamics in patients with primary liver cancer or other solid tumors (such as melanoma and renal cell carcinoma) and in patients with hematologic malignancies (like lymphoma and multiple myeloma) [120]. Preceding *in vitro* and *in vivo* studies in different cancer models, such as hepatocellular carcinoma, showed inhibition of tumor cells *in vitro*, efficient delivery of the lipid nanoparticles to the liver, significant tumor regression, prolonged survival of treated mice carrying this type of tumor, and even some tumor-free mice after treatment [120].

Mirna Therapeutic, an American company in miRNA therapeutics, developed the first miRNA replacement therapy for application in cancer clinical trials, using miR-34a mimics (MRX34) incorporated into a lipid-based nanoparticle formulation called SMARTICLES [120]. This miRNA-based product is in a Phase I clinical trial in patients with primary liver cancer or other solid tumors (such as melanoma and renal cell carcinoma) and in patients with hematologic malignancies (like lymphoma and multiple myeloma) [120]. Preceding *in vitro* and *in vivo* studies in different cancer models, such as hepatocellular carcinoma, showed inhibition of tumor cells *in vitro*, efficient delivery of the lipid nanoparticles to the liver, significant tumor regression, prolonged survival of treated mice carrying this type of tumor, and even some tumor-free mice after treatment [120].

Although no miRNA-based gene therapy clinical trial has yet been approved for pancreatic cancer, the remarkable scientific data obtained with several miRNA-based therapeutic strategies, including with miR-34a mimics, both *in vitro* and in preclinical studies, envision an auspicious future for miRNA-based therapies in PDAC. Despite miRNA gene therapy in cancer has still a long way to go, miRNA-based therapeutics present a broad number of options to be successfully used in a clinical context.

## 6. Conclusions and Future Perspective

MicroRNAs represent a fine-tuning in molecular pathways, more than massive modulators in cell physiologic events. Nevertheless, they are of great importance for the regulation of normal cell functions. Since their discovery, many studies had focused on a one-to-one relationship between miRNA and genes. Over the time, this view was quickly surpassed by novel discoveries which illustrated a much more complex network between these two players and an intricate regulation of biological systems sustained by differential miRNA expression pattern [125].

Understanding the full extent of miRNAs functional activity, from their biology to molecular properties, has allowed the development of bioinformatics tools that make use of computational algorithms, based on specific base-pairing rules and cross-species conservation requirements, to predict the targeting of a given mRNA by a specific miRNA [126]. Consequently, a huge amount of data has emerged from the interface of the bioinformatics and biologic system studies, leading to the development of miRnomics as a new field in science and to the creation of several miRNA databases for the scientific community [127,128]. Nevertheless, bioinformatics tools may deliver false positive miRNA-mRNA correlation, as the biological context of these RNA molecules interactions are not taken in consideration or poorly estimated, enhancing the need for an integrative approach with experimental studies in order to establish a comprehensive view on this computational predictions [125].

Acknowledging that miRNA present two extremely important features in terms of genome expression regulation, which are multiplicity and cooperativity, where one miRNA can target more than one gene (multiplicity), and one gene can be controlled by more than one miRNA (cooperativity) [129], the hypothesis of obtaining a wide control over cell tumorigenic properties through the modulation of a small group of miRNAs, with high multiplicity scores, seems a thrilling opportunity to boost miRNA therapeutics. The relevance of these small regulatory RNA molecules in cancer had been foreseen as potential diagnostic and prognostic molecular markers, or for the identification of tumor subtypes according to their miRNA profile. However, miRNAs are also seen as highly promising therapeutic agents, mostly due to the ability of each single miRNA to target several crucial pathways involved in tumorigenesis maintenance.

Nevertheless, despite the enormous therapeutic potential of these molecules, successful clinical application of miRNA-based strategies remains dependent on the generation of safe and efficient delivery systems capable of overcoming the numerous biological barriers associated to this process, so that efficient uptake of nucleic acids into target cells can be achieved. Much work has been devoted to this requirement and many different approaches for tumor delivery of miRNAs have been developed in order to allow miRNA-based therapeutic approaches being successfully applied in a clinical context for the treatment of pancreatic cancer and others cancer diseases.

On the other hand, recent developments in new cancer therapeutics have shown that combination of antitumor gene therapy strategies, such as those involving AMOs or miRNA mimics, with low amounts of chemotherapeutic agents could result in a synergistic and strong antitumor effect in different tumor models, including PDAC [53,130]. These data constitute an evidence of the remarkable opportunity to efficiently combine miRNA-based therapeutic strategies with chemotherapeutic regiments as a novel approach with great potential for future cancer therapy.

## Figures and Tables

**Table 1:** Experimental chemotherapeutic regiments for pancreatic ductal adenocarcinoma (PDAC).

Agent/Year of Publication	Arm Comparision	Response Rate (%)	Median Overall Survival (Months)	Observations
Fluorouracil (5-FU) 1969 [14]	fluorouracil (5-FU) combined with radiotherapy	-	10.4	Increased incidence of side effects when compared to radiotherapy alone
	radiotherapy	-	6.3	
gemcitabine 1997 [15]	gemcitabine	23.8	5.65	Higher mitigation of disease-related symptoms when compared to 5-FU
	fluorouracil (5-FU)	4.8	4.41	
FOLFIRINOX 2011 [16]	FOLFIRINOX	31.6	11.1	Increased degree of side effects when compared to gemcitabine
	gemcitabine	9.4	6.8	
Nab-paclitaxel 2013 [17]	Nab-paclitaxel + gemcitabine	23.0	8.5	Higher clinical response when compared to gemcitabine
	gemcitabine	7	6.7	

**Table 2:** Most frequently deregulated microRNAs in PDAC.

MicroRNA	Expression Profile	Function	Demonstrated Targets in PDAC
hsa-miR-10b	up-regulated	invasiveness	TIP30 [44]
hsa-mir-100	up-regulated	cell proliferation and metastization	IGFR1 [45]
hsa-miR-155	up-regulated	cell proliferation, invasiveness and migration ability	TP53INP1 [46]; SOCS1 [47];
hsa-miR-196a	up-regulated	cell proliferation and invasiveness	NFKBIA [48]; ING5 [49]
hsa-miR-21	up-regulated	metastization, cell proliferation and chemotherapeutic resistance	PDCD4 [50,51]; TIMP3 [50]; PTEN [51],
hsa-miR-221/222	up-regulated	cell proliferation, survival, migration, invasiveness, metastization	PDGF [52]; p27^Kip1^ [53,54]; p57^kip2^, PUMA [54]; TIMP-2 [55]
hsa-miR-23a	up-regulated	cell proliferation	APAF-1 [56]
hsa-miR-148a	down-regulated	metastasis suppression	CDC25B [57]; CCKBR [58]; Bcl-2 [58]; DNMT1 [59]
hsa-miR-217	down-regulated	suppression of cell growth	KRAS [60]; SIRT1 [61]
hsa-miR-34a	down-regulated	cell cycle arrest, apoptosis, suppression of tumor cell migration	E2F3; Bcl-2; c-MYC; Cyclin D1 [62]; Notch-1 [63]
hsa-miR-375	down-regulated	glucose homeostasis, cell cycle arrest	PDPK1 [64,65]

hsa (human miRNA); TIP30 (Tat-interacting protein 30); IGFR1 (insulin-like growth factor 1 receptor); TP53INP1 (tumor protein 53-induced nuclear protein 1); SOCS1 (suppressor of cytokine signaling 1); NFKBIA (nuclear factor κB-inhibitor α); ING5 (inhibitor of growth protein 5); PDCD4 (programmed cell death 4); TIMP3 (metallopeptidase inhibitor 3); PTEN (phosphatase and tensin homolog); PDGF (platelet-derived growth factor); p27^Kip1^ (cyclin-dependent kinase inhibitor 1B); p57^Kip2^ (cyclin-dependent kinase inhibitor 1C); PUMA (p53 upregulated modulator of apoptosis); TIMP-2 (metallopeptidase inhibitor 2); APAF-1 (apoptotic protease activating factor 1); CDC25B (cell division cycle 25 B); CCKBR (cholecystokinin-B receptor); Bcl-2 (B cell lymphoma 2); DNMT1 (DNA (cytosine-5-)-methyltransferase 1); KRAS (kirsten rat sarcoma viral oncogene homolog); SIRT1 (sirtuin 1); E2F3 (E2F transcription factor 3); c-MYC (v-myc avian myelocytomatosis viral oncogene homolog); PDPK1 (3-phosphoinositide-dependent protein kinase 1).

**Table 3:** MicroRNA-based antitumor strategies in preclinical and clinical trials.

Trial		Delivery System	Target MicroRNA	Type of Cancer	Reference
Preclinical		Cationic liposome-based system	miR-29b	lung cancer	[114]
		Exosomes based nanosystem	miR let-7	breast cancer	[115]
		Oncolytic Adenovírus	miRNA-34a	hepatocellular carcinoma	[116]
		Lipid-based nanosystem	miR-34a and miR-143/145 cluster	pancreatic cancer	[117]
		Nanocomplexes	miR-34a	pancreatic cancer	[62]
Clinical	Phase II	Miravirsen (Santaris Pharma)	miR-122 inhibitor	hepatitis C	[118,119]
	Phase I	MRX34 (Mirna Therapeutics)	miR-34a mimics	advanced solid tumors	[120]
